# Supplementary material for: Reactive astrocytic S1P3 signaling modulates the blood–tumor barrier in brain metastases
Source: Nat Commun. 2018 Jul 13;9:2705. doi: 10.1038/s41467-018-05030-w (PMC6045677; doi:10.1038/s41467-018-05030-w)
Supplement: Supplementary file 1 — Supplementary Information [file 41467_2018_5030_MOESM1_ESM.pdf]

Supplementary information

Reactive astrocytic S1P3 signaling modulates the blood-tumor barrier in brain metastases

Gril *et al*.

Supplementary Table 1: Antibodies used in the study

Antibody	Catalog number	Company	PMID	Concentration/Dilution
CCL2	MAB679	R&D	27666332	2 μg mL^{-1}
CD31	550274	BD Biosciences	7956830	1:500
CXCL1	MAB276	R&D	24872191	3 μg mL^{-1}
Cytokeratin	M0821	Dako	24829032	1:100
Epha5	GTX25398	GeneTex		1:50
GABA Aα1	75-136	NeuroMab	26685158	1:50
GABA Aγ2	224 003	Synaptic systems	22286174	1:50
GFAP	MAB360	Millipore	25808087	1:10,000
GM-CSF	MAB215	R&D	24562309	1 μg mL^{-1}
IL-6	MAB206	R&D	28930684	1 μg mL^{-1}
IL-8	MAB208	R&D	25952647	1 μg mL^{-1}
Ki67	M7240	Dako	29084952	1:100
NeuN	MAB377	Millipore	25915831	1:50
NG2	AB5320	Millipore	26439639	1:100
S1P3	NBP1-00789	Novus		1:100
S1P3	LS-B2155	LSBio	20648639	1:100
VE-cadherin	ab33168	Abcam	28166726	1:100
ZO-1	61-7300	ThermoFisher	28508859	1:100
αSMA	A2547	Sigma-Aldrich	21258411	1:100

a

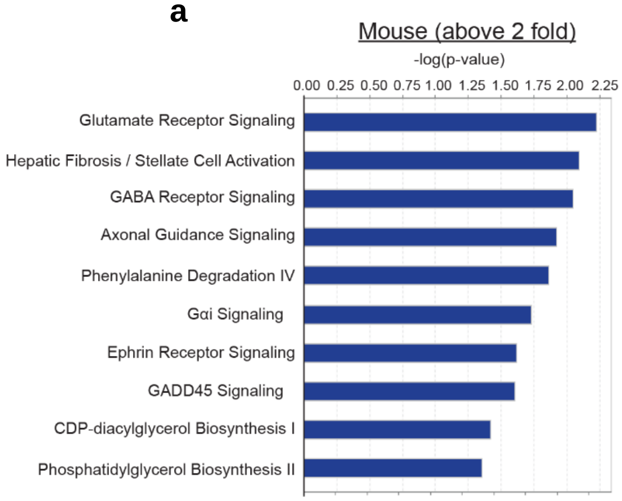

Glutamate Receptor Signaling	GNG2,SLC1A3,GRIP1,GRIN3A
Hepatic Fibrosis / Hepatic Stellate Cell Activation	COL19A1,COL5A3,COL6A1,COL4A1,IGFBP3,CCL5,IL1RAP
GABA Receptor Signaling	GABRG1,GABRA1,GABRA2,GABRA3
Axonal Guidance Signaling	GNG4,SHH,PLXNC1,KALRN,GNG2,BAIAP2,UNC5D,DCC,EPHA5,PLXNA2,EPHA3,SLIT2
Phenylalanine Degradation IV (Mammalian, via Side Chain)	MAOB,SLC27A2
Gαi Signaling	S1PR3,GNG4,NPR3,PTGER3,GNG2
Ephrin Receptor Signaling	GNG4,KALRN,GNG2,EPHA5,EPHA3,GRIN3A
GADD45 Signaling	CCND1,CCNB1
CDP-diacylglycerol Biosynthesis I	MBOAT1,MBOAT7
Phosphatidylglycerol Biosynthesis II (Non-plastidic)	MBOAT1,MBOAT7

b

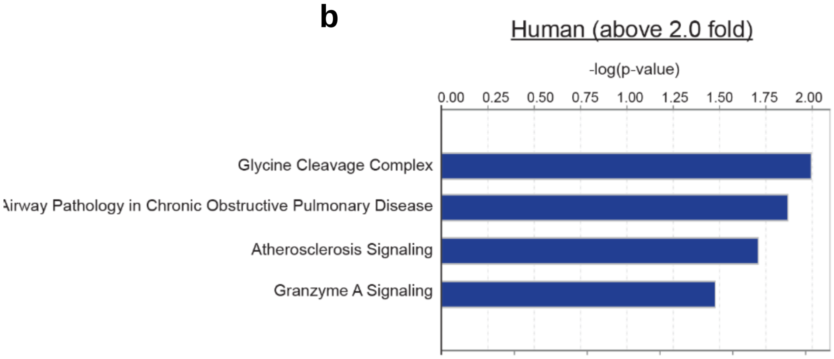

Glycine Cleavage Complex	OCA2
Airway Pathology in Chronic Obstructive Pulmonary Disease	MMP1
Atherosclerosis Signaling	SERPINA1,MMP1
Granzyme A Signaling	GZMA

Supplementary Fig. 1. Ingenuity® pathway analysis of canonical pathways in datasets comparing highly permeable versus poorly impermeable lesions. Significantly affected canonical pathways in datasets comparing highly permeable versus poorly impermeable lesions in mouse **(a)** and human **(b)** array datasets. Both datasets comprised only the genes those were up- or down-regulated 2-fold or above. The significance was calculated by Fisher's exact test (right-tailed). Data were analyzed using Ingenuity Pathways Analysis (Ingenuity® Systems, www.ingenuity.com). List of genes involved is tabulated below the graphs.

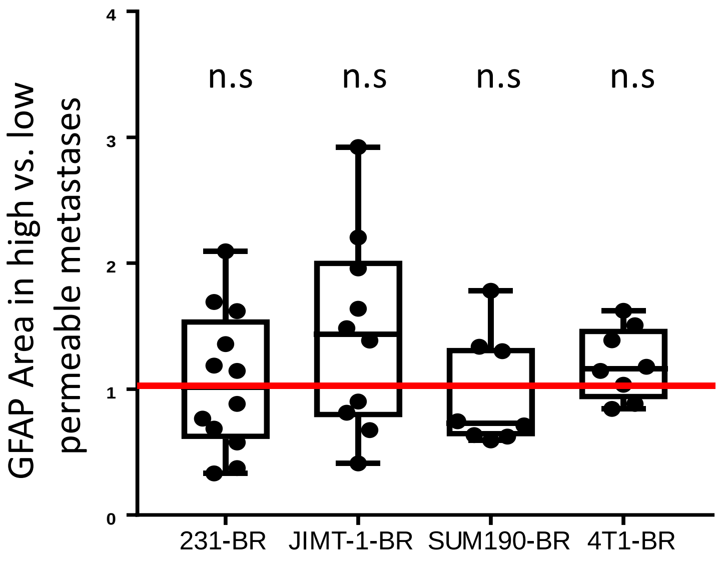

Supplementary Fig. 2 Quantification of GFAP activated astrocytes in the four metastatic models. The quantification of GFAP activated astrocytes for the 231-BR (n=12) and JIMT-1-BR (n=10) models were published in Lyle *et al*[1]. Only the quantification of GFAP activated astrocytes in the SUM190-BR (n=8) and the 4T1-BR (n=8) models results from new cohorts of mice. In the four models, GFAP+ activated astrocytes are present randomly in and around both highly and poorly permeable lesions, i.e., there is no correlation to pattern of permeability. The medians are 1.014, 1.44, 0.729, and 1.2 for 231-BR, JIMT-1-BR, SUM190-BR, and 4T1-BR respectively. Each dot represents one mouse and graphs represent the median, the interquartile range and the min/max values. Two-tailed Mann-Whitney statistical analysis. ns: non-significant.

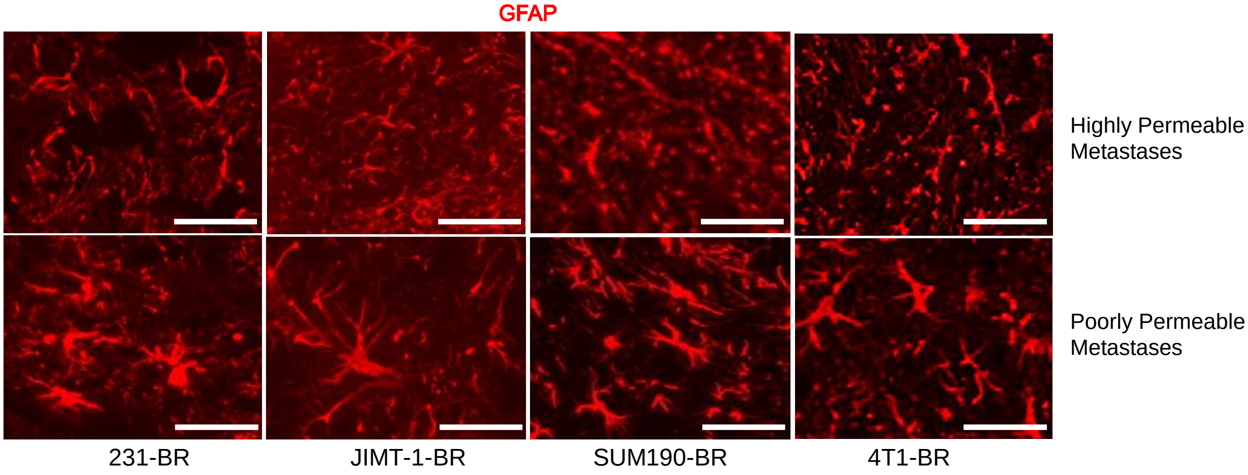

Supplementary Fig. 3 Morphological characteristics of GFAP activated astrocytes in the four metastatic models. Mice were injected with brain-tropic cell lines and brain metastases permitted to form. Before necropsy, mice were injected with Texas Red Dextran (TRD), which was permitted to circulate before perfusion. Sections from mouse brains harboring experimental metastases from the indicated model systems were assayed for TRD permeability and the adjacent section stained for GFAP. GFAP activated astrocytes appeared thinner and more punctiform in the highly permeable lesions, while in the poorly permeable they maintained a more traditional activated shape with large cellular body and defined extensions. Scale bars = 50 µm.

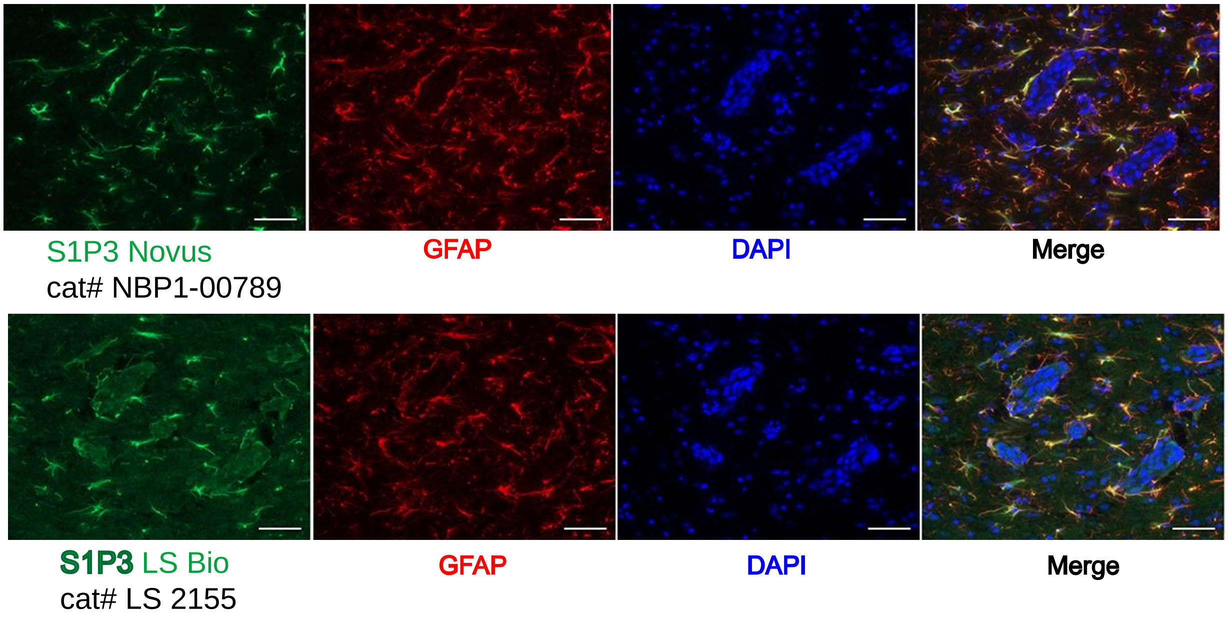

Supplementary Fig. 4 S1P3 immunostaining. Mice containing experimental brain metastases of breast cancer were necropsied and the brains sectioned and stained as indicated. Two different commercially available antibodies against S1P3 gave the same staining, highlighting the activated astrocytes surrounding the metastasis lesions (on the top panel antibody from Novus and on the bottom panel antibody from Lifespan Bioscience). Scale bars = 50 μm.

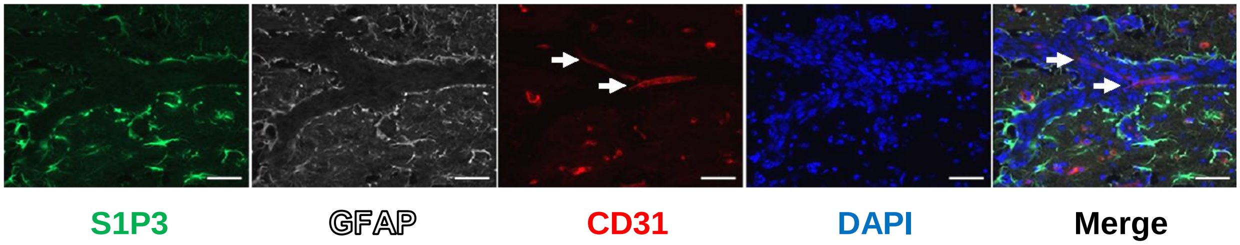

Supplementary Fig. 5 S1P3 expression is not detected in the vasculature. Mice containing experimental brain metastases of breast cancer were necropsied and the brains sectioned and stained as indicated. S1P3+ activated astrocytes do not line the blood vessels inside the metastatic clusters (BTB). The blood vessels inside the metastases are shown by CD31 staining identified by the white arrows. Scale bars = 50 µm.

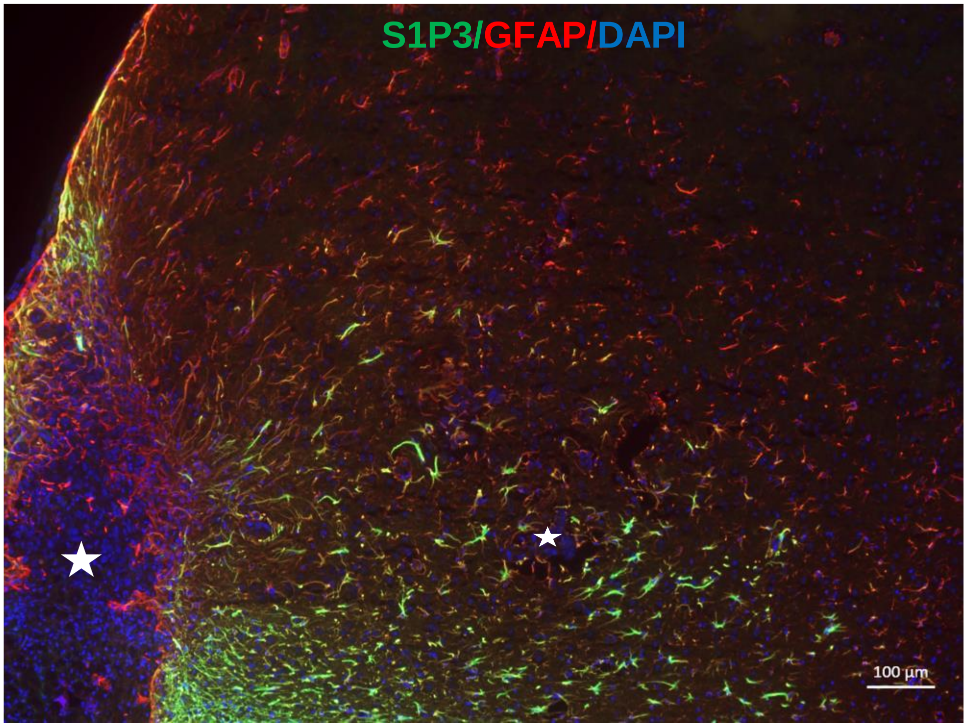

Supplementary Fig. 6 S1P3 astrocytes are near the metastatic lesions. Mice containing experimental brain metastases of breast cancer were necropsied and the brains sectioned and stained as indicated. Low magnification shows that the S1P3 astrocytes are the ones closer to the metastatic lesions (marked by white stars) in the neuroinflammation region. Further away from the lesion (top right corner), the population of activated astrocytes visibly decreased, and S1P3 staining is absent. Scale bars = 100 µm.

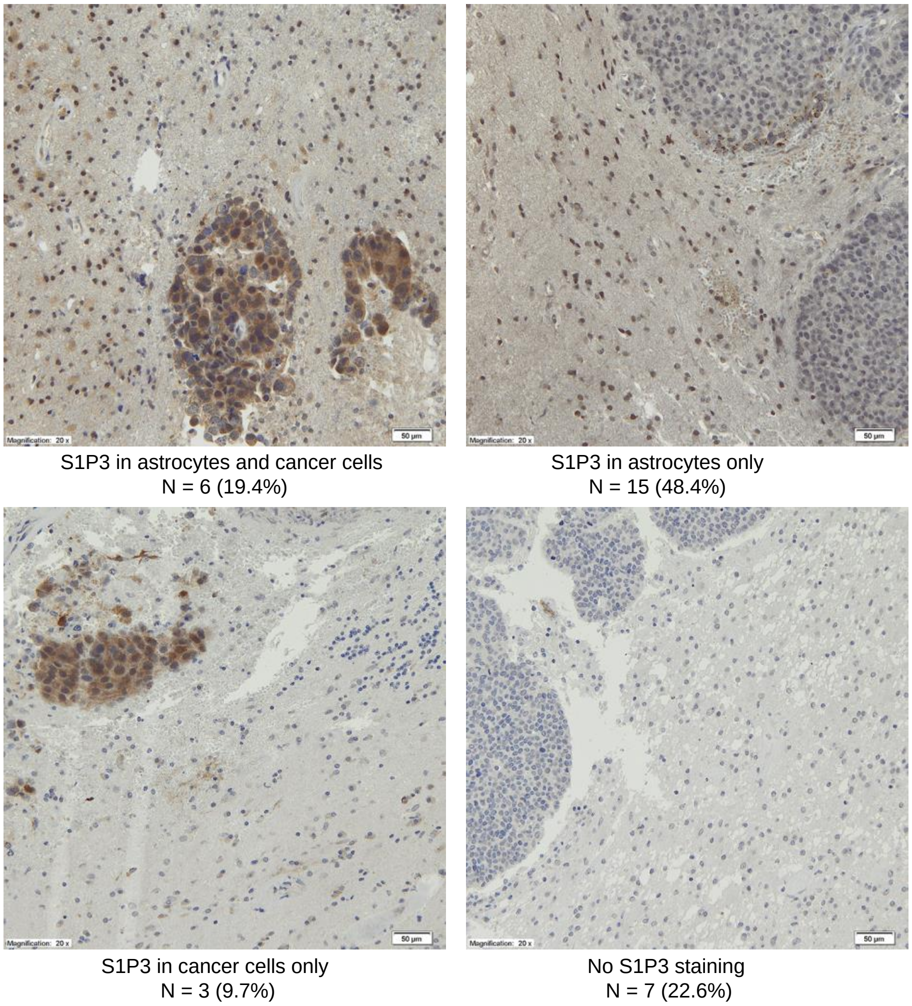

Supplementary Fig. 7 S1P3 staining of formalin fixed, paraffin-embedded clinical samples of brain metastases. Thirty-one brain metastasis samples from patients were stained for S1P3 and analyzed by a pathologist. Four patterns of staining were identified and representative pictures are presented. For each staining pattern, the number of samples is indicated with the percentage in parenthesis. Scale bars = 50 µm.

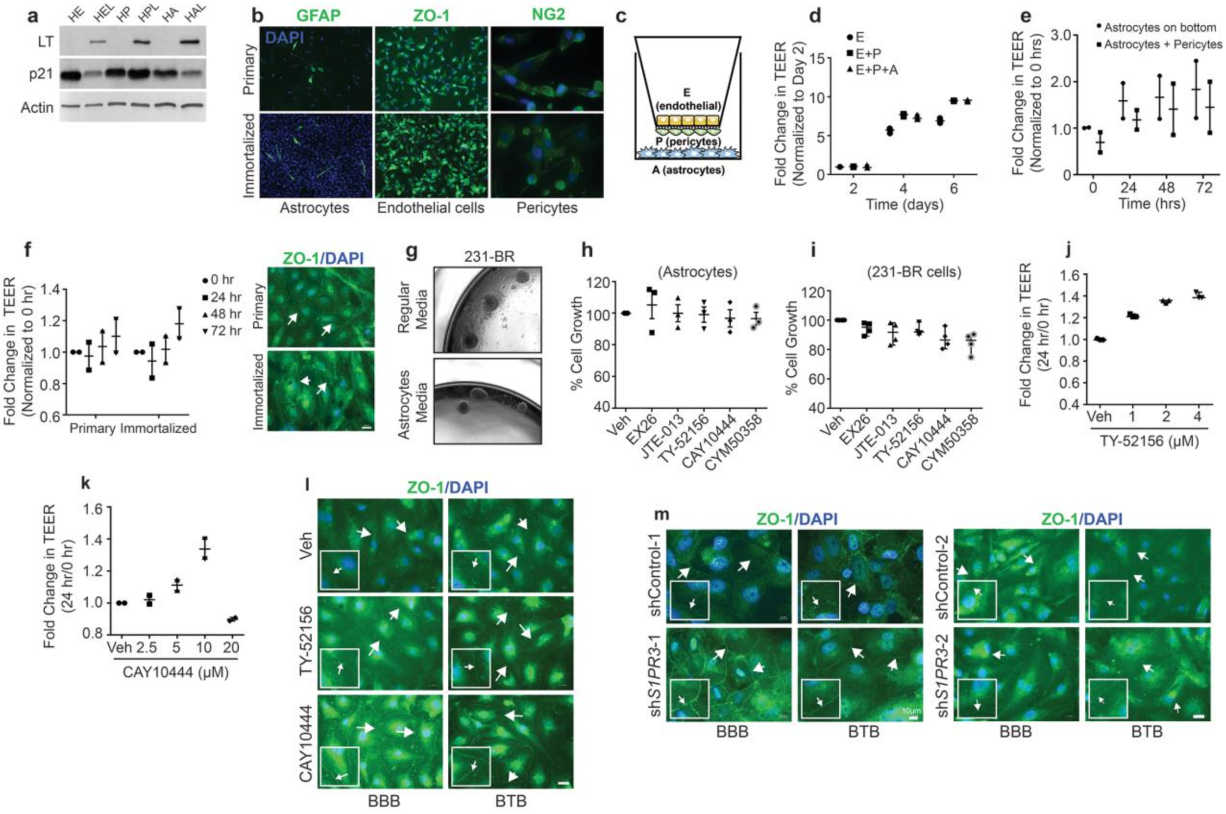

Supplementary Fig. 8 Validation of *in vitro* BBB/BTB assays. *In vitro* cultures of endothelial cells, pericytes and astrocytes have been previously reported[2-6]. Previous studies have shown that using immortalized endothelial cells to be a feasible alternative for primary cells[7]. By stably overexpressing SV40 Large T (SV40-LT) antigen using lentiviruses, we generated immortalized endothelial cells (HEL), pericytes (HPL), and astrocytes (HAL). **a** Western blot shows over-expression of SV40-LT and reduced p21 upon stable overexpression of SV40-LT in immortalized cells (H- human, E-endothelial cells, P-pericytes, A-astrocytes, L- Large T). **b** Immortalized cells retained parental phenotypes as shown by continued expression of GFAP (astrocytes), ZO-1 (endothelial cells), and NG2 (pericytes). **c** Schematic of *in vitro* BBB culture containing endothelial cells, pericytes, and astrocytes. **d** Addition of all three cell types yielded consistent TEER values (*n*=2). **e** TEER measurements showed mixing astrocytes with pericytes showed reduced TEER (*n*=2); astrocytes were therefore retained in the bottom on wells. **f** Functional comparison of primary and immortalized cultures. ZO-1 expression on the endothelial membrane (right panel, arrows). **g** Triple-negative, brain-tropic 231-BR cell spheres used in BTB assays. **h, i** Antagonists to S1P receptors were not cytotoxic using Alamar blue cell viability reagent (#DAL1100, ThermoFisher Scientific), according to manufacturer's protocol on both immortalized astrocytes (*n*=3) (**h**) and 231-BR (*n*=4) (**i**) cells. Dose response of TY-52156 (**j**) and CAY10444 (**k**) to TEER (*n*=3, *n*=2, respectively). S1P3 inhibitors (**l**) or sh*S1PR3* knockdown in astrocytes (**m**) resulted in decreased ZO-1 staining on endothelial cells (white arrows). Scale bars are 10 μm or 20 μm as indicated. The bars represent median values from independent transwell BBB/BTB cultures (*n*) from multiple experiments. Error bars indicate 95% confidence interval.

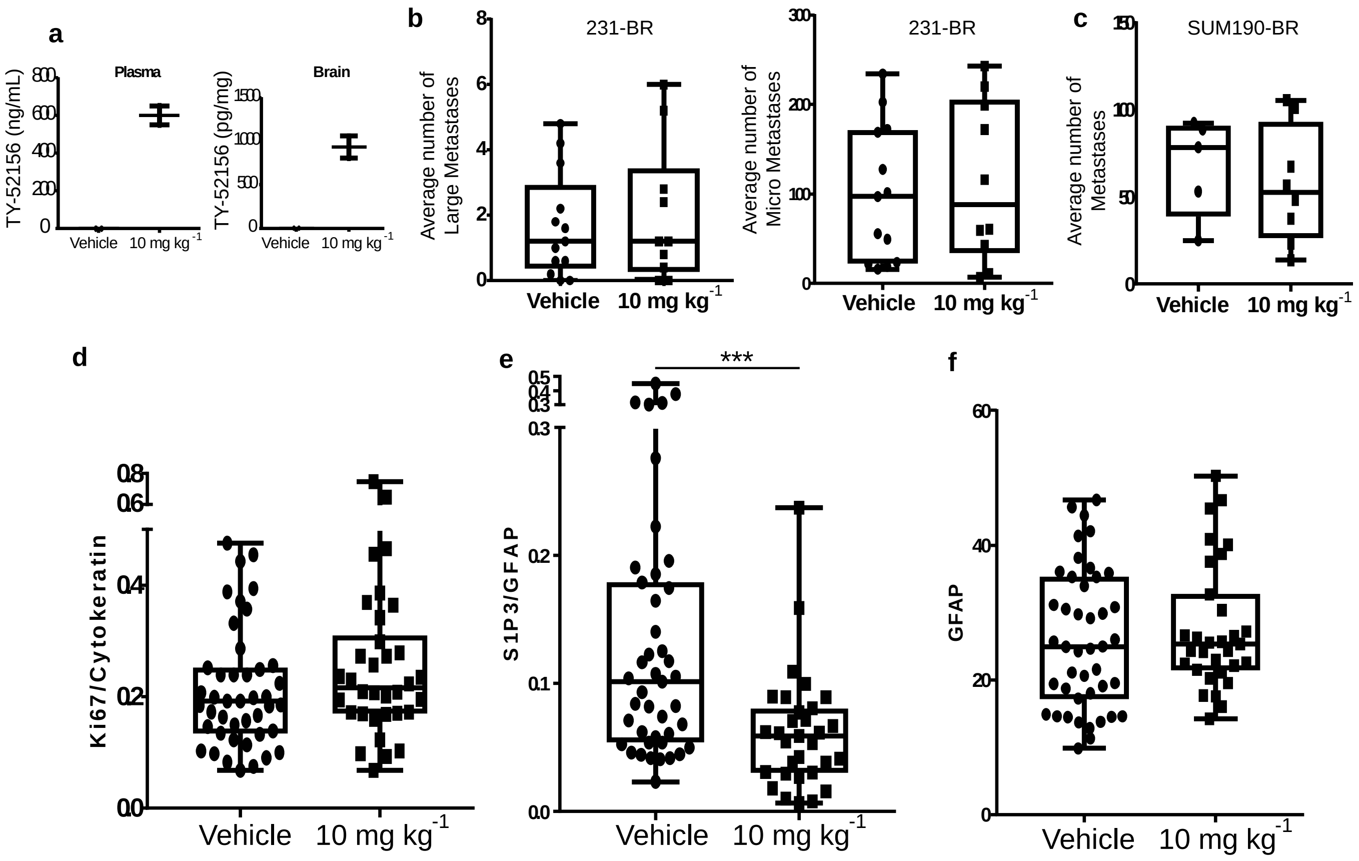

Supplementary Fig. 9 Effect of TY-52156 on the 231-BR brain metastasis models. **a** PKmeasurements of TY-52156 in plasma and blood (taken at the end point) of two animals treated with either vehicle or 10 mg kg^{-1} TY-52156, demonstrating uptake of compound in the brain. **b** Large metastases (≥300 µm) and micrometastases (<300 µm in a single dimension) were counted in H&E stained sections from the 231-BR model. TY-52156 had no effect on the number of metastases: vehicle median = 1.2, IQR = 0.4-2.9, *n*=11; 10 mg kg^{-1} TY-52156 median = 1.2, IQR = 0.3-3.4, *n*=8; (non-significant) for large metastases, and vehicle median = 97.4, IQR = 22.9-170.8; 10 mg kg^{-1} TY-52156 median = 88.4, IQR = 34.7-204.5 (non-significant) for micrometastases. **c** SUM190-BR lesions were counted in H&E stained sections. The number of metastases was not significantly different between vehicle and 10 mg kg^{-1} TY-52156: vehicle median = 78.4, IQR = 39.1-90.5 *n*=5; 10 mg kg^{-1} TY-52156 median = 52.5, IQR = 26.65-92.75, *n*=8; P = 0.436 (non-significant). **d** to **f,** Graphs represent metastatic clusters from 11 and 8 mice for vehicle and 10 mg kg^{-1} TY-52156, respectively. **d** Percentage of proliferative 231-BR metastatic cells at the endpoint: The ratio Ki67/cytokeratin in each metastasis was quantified by IF staining. TY-52156 had no effect on metastatic cell proliferation: Vehicle median = 0.191, IQR = 0.335-0.251; 10 mg kg^{-1} median = 0.216, IQR = 0.17-0.31; P = ns. **e** TY-52156 decreased S1P3 expression in GFAP+ astrocytes. (vehicle median = 0.101, IQR = 0.054-0.18; 10 mg kg^{-1} TY-52156 median = 0.059, IQR = 0.007-0.08; P < 0.001). **f** TY-52156 had no effect on the total number of activated astrocytes (vehicle median = 24.92, IQR =17.25-35.3; 10 mg kg^{-1} TY-52156 median = 25.32, IQR = 21.51-32.71; P = ns). Graphs represent the median, the interquartile range and the min/max values. Two-tailed Mann-Whitney statistical analysis; ***P < 0.001.

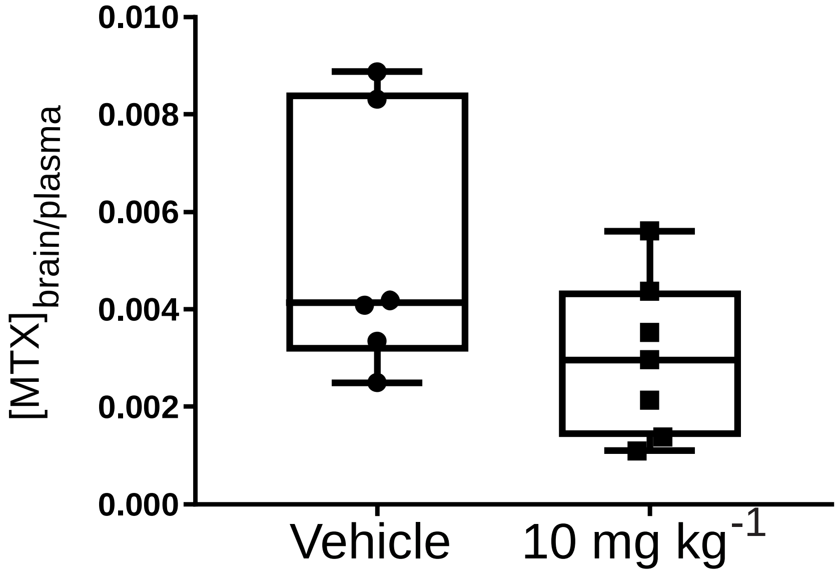

Supplementary Fig. 10 TY-52156 induces a trend in decreasing methotrexate brain uptake, by strengthening the blood-tumor barrier *in vivo*. Mice were injected with the 231-BR cancer cells. At day 25, when the metastases were developed, mice received vehicle or 10 mg kg^{-1} TY-52156 treatment b.i.d. by oral gavage, for four days (day 25th to 28th). On day 26th and 28th, all the mice received a dose of methotrexate (MTX) (50 mg kg^{-1}). On the 28th day, the last dose of TY-52156 was administrated 90 min before euthanasia and the last dose of MTX was administrated 30 min before euthanasia. At the end-point, mice were perfused for 10 min to remove the compound from the vasculature. Brain and blood were taken at necropsy to analyze MTX concentration via liquid chromatography tandem mass chromatography (LC–MS/MS). The median in vehicle was 4.139 $x10^{-3}$ (IQR = $3.14x10^{-3}$- 8.45 $x10^{-3}$, *n*=6) and decreased to 2.965 $x10^{-3}$ (IQR = $1.34x10^{-3}$- 4.38 $x10^{-3}$, *n*=7) after TY-52156 treatment. The difference was not statistically significant (*P* = ns, two-tailed Mann-Whitney test).

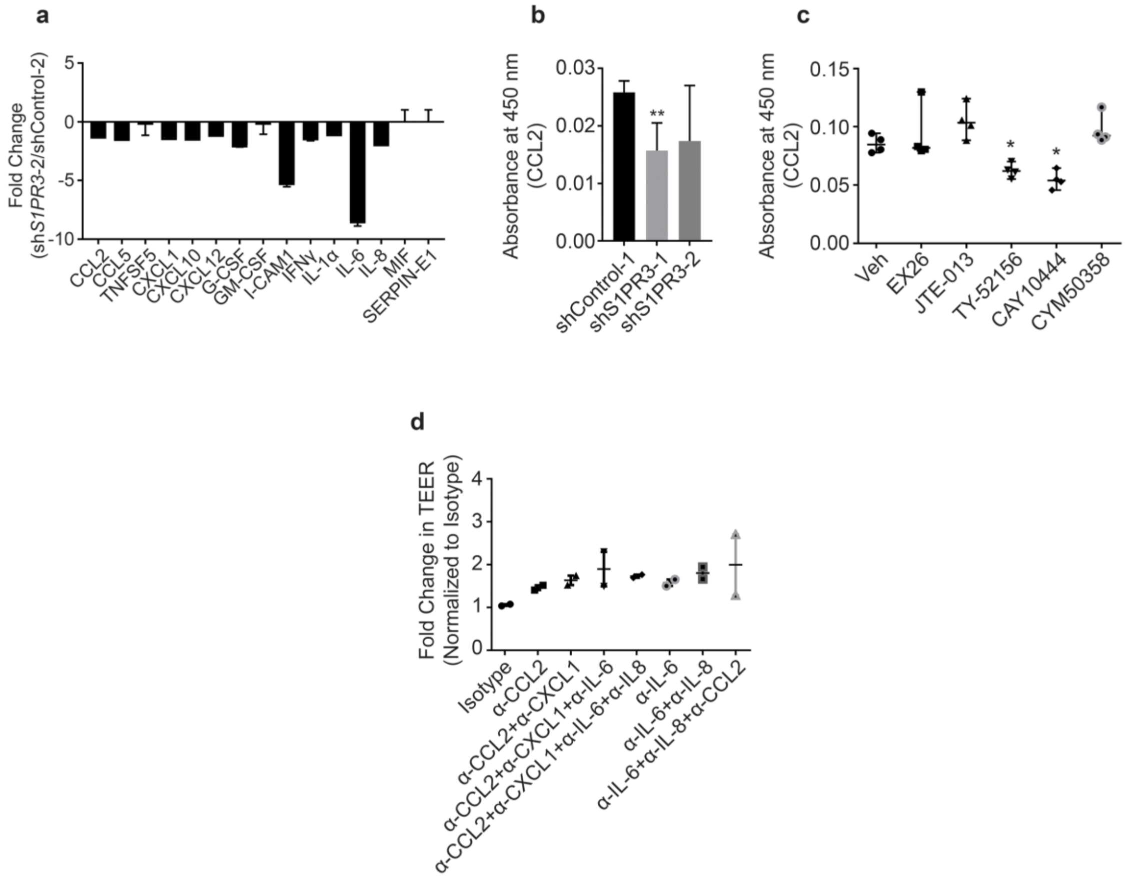

Supplementary Fig. 11 Knockdown of S1P3 in astrocytes reduced expression of secreted cytokines. **a** Serum-free supernatant from shControl-2 and sh*S1PR3*-2 astrocytes were compared for expression of 36 known cytokines and chemokines using commercial human cytokine array. The detectable spots were quantified using ImageJ and graph was plotted for fold-change. **b** Relative CCL2 levels as assessed by ELISA assay in cell supernatant of shControl-1, sh*S1PR3*-1, and sh*S1PR3*-2 astrocytes. Knockdown of S1P3 in astrocytes resulted in reduction of secreted CCL2 levels (n=6) validating cytokine array results for sh*S1PR3*-1 and -2 cells. Kruskal-Wallis test and Dunn's multiple comparison test were performed. **c** Relative CCL2 levels in cell supernatant of astrocytes treated with S1P receptor antagonists. Inhibition of S1P3 (using TY-52156 and CAY10444) but not S1P1, S1P2, or S1P4 resulted in significant reduction in secreted CCL2 levels (n=4). **d** Combinatorial treatment using various cytokine neutralizing antibodies did not enhance the TEER to a significant level (n=2). Graphs represent medians and the error bars represent 95% confidence interval. *P <0.05, **P <0.01.

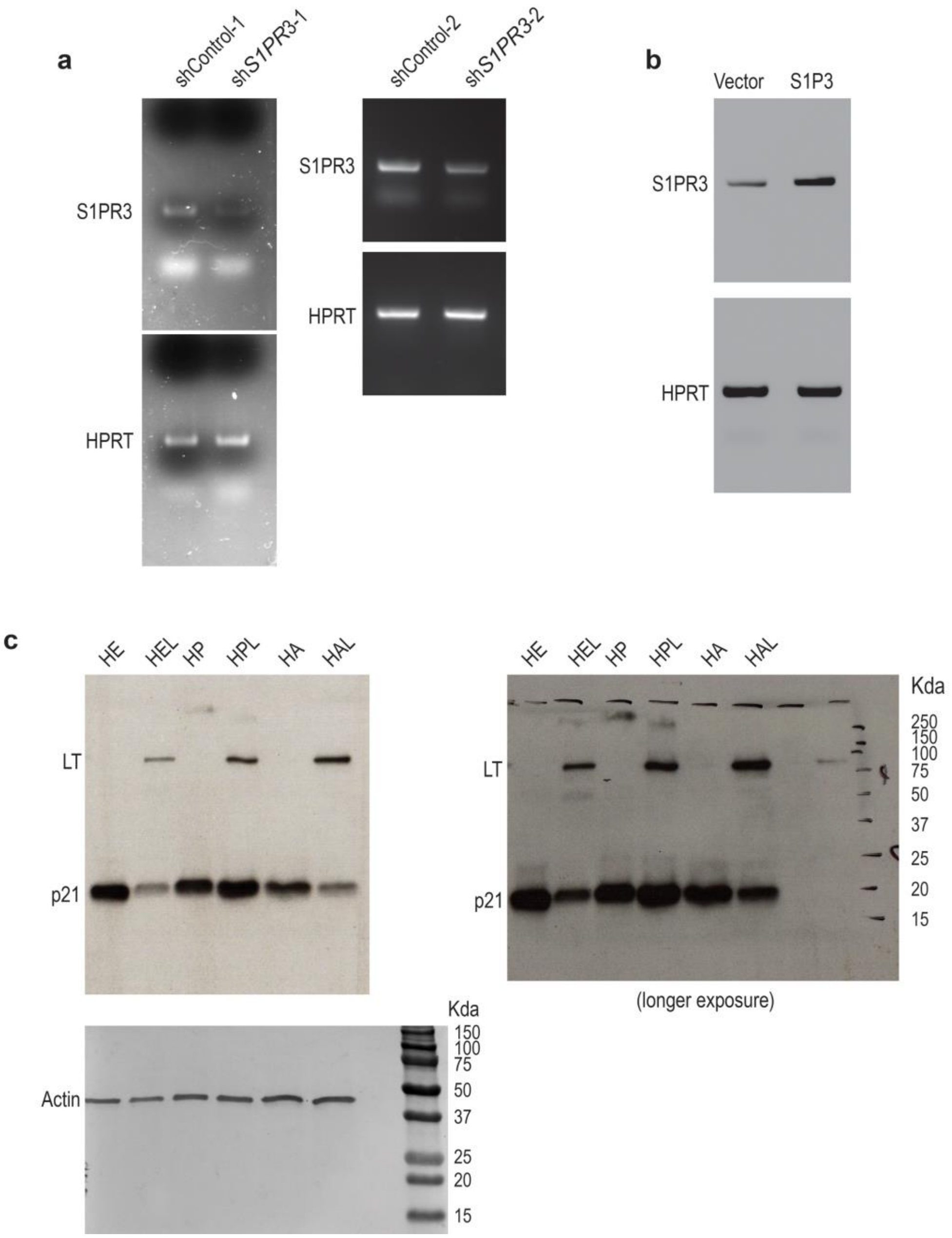

Supplementary Fig. 12 Original images of RT-PCR gels and Western blots used in the study. **a** Agarose gel image shows S1PR3 bands and loading control HPRT bands for RT-PCR results presented in Fig. 6a. **b** Agarose gel images show S1PR3 bands and loading control HPRT bands for RT-PCR results presented in Fig. 9a. **c** Western blot gels show bands for LT, p21, and loading control actin, presented in Supplementary Fig. 8a. The image on the right shows longer exposure.

Supplementary References

1. Lyle, L. T. *et al.* Alterations in Pericyte Subpopulations Are Associated with Elevated Blood-Tumor Barrier Permeability in Experimental Brain Metastasis of Breast Cancer. *Clin Cancer Res* **22**, 5287-5299, doi:10.1158/1078-0432.CCR-15-1836 (2016).
2. Bowman, P. D. *et al.* Primary culture of capillary endothelium from rat brain. *In Vitro* **17**, 353-362 (1981).
3. Bowman, P. D., Ennis, S. R., Rarey, K. E., Betz, A. L. & Goldstein, G. W. Brain microvessel endothelial cells in tissue culture: a model for study of blood-brain barrier permeability. *Ann Neurol* **14**, 396-402, doi:10.1002/ana.410140403 (1983).
4. Panula, P., Joo, F. & Rechardt, L. Evidence for the presence of viable endothelial cells in cultures derived from dissociated rat brain. *Experientia* **34**, 95-97 (1978).
5. Arthur, F. E., Shivers, R. R. & Bowman, P. D. Astrocyte-mediated induction of tight junctions in brain capillary endothelium: an efficient in vitro model. *Brain Res* **433**, 155-159 (1987).
6. Nakagawa, S. *et al.* A new blood-brain barrier model using primary rat brain endothelial cells, pericytes and astrocytes. *Neurochem Int* **54**, 253-263, doi:10.1016/j.neuint.2008.12.002 (2009).
7. Weksler, B. B. *et al.* Blood-brain barrier-specific properties of a human adult brain endothelial cell line. *FASEB J* **19**, 1872-1874, doi:10.1096/fj.04-3458fje (2005).
