# Supplementary material for: Reactive astrocytic S1P3 signaling modulates the blood–tumor barrier in brain metastases
Source: Nat Commun. 2018 Jul 13;9:2705. doi: 10.1038/s41467-018-05030-w (PMC6045677; doi:10.1038/s41467-018-05030-w)
Supplement: Supplementary file 3 — Description of Additional Supplementary Files [file 41467_2018_5030_MOESM3_ESM.pdf]

Description of Additional Supplementary Files

File Name: Supplementary Data 1
Description: Mouse microarray

File Name: Supplementary Data 2
Description: Human microarray

File Name: Supplementary Data 3
Description: S1P3 and GFAP staining in the human specimens
